# Antioxidant and Phytochemical Studies of 31 Cowpeas (*Vigna unguiculata* (L. Walp.)) Genotypes from Burkina Faso

**DOI:** 10.3390/foods7090143

**Published:** 2018-09-03

**Authors:** Pierre Alexandre Eric Djifaby Sombié, Moussa Compaoré, Ahmed Yacouba Coulibaly, Jeremy Tinga Ouédraogo, Jean-Baptiste De La Salle Tignégré, Martin Kiendrébéogo

**Affiliations:** 1 National Center of Scientific Research and Technology, Institute of Environment and Agricultural Research, Crop Production Department, 01 P.O. Box 476 Ouagadougou, Burkina Faso; jouedraogo5@hotmail.com (J.T.O.); racinetignegre@yahoo.com (J.-B.D.L.S.T.); 2 Laboratory of Biochemistry and Chemistry Applied (LABIOCA), University of Ouaga I Professor Joseph Ki-Zerbo, 09 P.O. Box 848 Ouagadougou, Burkina Faso; mcompaore_3@yahoo.fr (M.C.); coulahmede@yahoo.fr (A.Y.C.); martinkiendrebeogo@yahoo.co.uk (M.K.); 3 Unit of formation and Research in Sciences and Technology, University Norbert Zongo, P.O. Box 376 Koudougou, Burkina Faso

**Keywords:** *Vigna unguiculata*, phenolic content, antioxidant activities, genotype

## Abstract

Antioxidant compounds of dietary plants have been widely studied because of their bioactive properties. The objective of this research study was to analyse the health enhancing attributes of 31 cowpeas varieties from Burkina Faso. Significant variations were observed in the phenolic content as well as the antioxidant and anti-lipid peroxidation activities amongst the cowpea varieties. Pearson correlation coefficient analysis showed that the ferric reducing ability (*r* = 0.954) and anti-lipid peroxidation (*r* = 0.616) were positively correlated with the total phenolic content. A significant relationship between cowpea ferric reducing ability and anti-lipid peroxidation (*r* = 0.64) was also revealed. However, nitric oxide scavenging potential was found not to be related to its total phenolic and total flavonoid content. 2,2-diphenyl-1-picrylhydrazyl (DPPH) and hydroxyl radicals scavenging potentials were not correlated with the total flavonoid content. The pigmented seeds of cowpea varieties possess higher total phenolic, total flavonoid content, ferric reduction ability, and anti-lipid peroxidation activities than the colourless ones. The results obtained from this study suggest that Burkina Faso cowpea cultivars are rich in phenolic compounds and have significant antioxidant and anti-lipid peroxidation activities. Consumption of the cowpea, particularly of coloured cowpea seed varieties, should be beneficial for chronic human diseases prevention.

## 1. Introduction

*Vigna unguiculata* L. Walp. (cowpea) is a widely cultivated legume in Asia, Central and South America, and Africa [1]. It is a staple food that provides for human nutrition large amounts of proteins, carbohydrates, dietary fibres, vitamins of the β complex, essential minerals, a small quantity of lipids, and has lower levels of anti-nutritional factors [2,3,4]. Cowpea seeds also contain bioactive compounds that may be beneficial to human health [2,5]. Phenolic compounds, the most important group of bioactive compounds in cowpea, are concentrated in the seed coat and have the potential to protect the body against chronic diseases [4,6]. The phenolic compounds are responsible for most of the coloration observed in diverse cowpea seeds. The major phenolic compounds present in leguminous seeds, particularly in cowpea, are the phenolic acids and flavonoids [6,7]. Flavonoids also play an important role in plant protection [2]. Phenolic compounds are believed to be responsible for the antioxidant and other health-promoting properties of cowpea [8]. Epidemiological studies have reported that the consumption of phenolic antioxidants-rich foods protects against human chronic non-communicable diseases such as cancer, ageing, diabetes, and cardiovascular disease [7]. Phenolic compounds act as scavengers of radicals, reducing agents, and metal ion chelators [7]. According to Kapravelou et al. [1], cowpea seeds exhibit beneficial health effects related to their antioxidant, hypoglycaemic, hypolipidaemic, and antihypertensive properties. Phenolic content composition and bioactive properties of cowpea can vary considerably depending on the cultivar. The objective of this study was to investigate the phenolic content (total phenolic and flavonoid) and antioxidant potentials of thirty-one cowpea varieties and to explore the relationship between phenolic content, antioxidant activities, and the colour of the seeds.

## 2. Materials and Methods

### 2.1. Plant Material

Cowpea (*Vigna unguiculata* (L. Walp.)) seeds of thirty-one (31) varieties were obtained from the genetic and biotechnology laboratory germplasm, Department of Crop Production, Institute of Environment and Agricultural Research (INERA), Center for Environmental, Agricultural and Training Research of Kamboinsé (CREAF-Kamboinsé), Ouagadougou, Burkina Faso.

### 2.2. Extraction Method

The dried seeds of cowpea varieties were ground to powder by using a coffee grinder. Seed powders (0.5 g each) of the cowpea varieties were extracted with 10 mL of acetone:water (80:20, *v*/*v*). The melange was shaken for 24 h and centrifuged at 4500 rpm for 15 min. The supernatant was used for the quantification of total phenolics, total flavonoids, and antioxidant and anti-peroxidation activities.

### 2.3. Phenolic Content

#### 2.3.1. Total Phenolic Content

The total phenolic content of cowpea seeds extracts was determined at 760 nm using gallic acid as reference compound, as described by Singleton et al. [9]. The total phenolics were expressed as mg of gallic acid equivalent per 100 g of dry seed weight (mg GAE/100 g dw).

#### 2.3.2. Total Flavonoid Content

The total flavonoid content of the cowpea seed extracts was determined at 415 nm using the method described by Arvouet-Grand et al. [10]. The total flavonoid content was determined on a quercetin calibration curve and expressed as mg of quercetin equivalents (QE) per 100 g of dry seed weight (mg QE/100 g seeds dw).

### 2.4. Antioxidant Activities

#### 2.4.1. Ferric Reducing Antioxidant Power (FRAP) Assay

The ability of the cowpea seed extracts to reduce iron (III) to iron (II) was measured at 700 nm following the procedure described by Sombié et al. [11]. Iron (III) reducing activity was determined as mg quercetin equivalents per 100 g of dry seed weight (mg QE/100 g of seeds dw).

#### 2.4.2. DPPH Radical Scavenging Activity

The ability of cowpea seeds extracts to scavenge the DPPH (2,2-diphenyl-1-picrylhydrazyl) radical was evaluated at 517 nm as described by Sombié et al. [11]. The means of three values were obtained, expressed as mg of ascorbic acid equivalent per 100 g of dry seeds weight (mg AAE/100 g seeds dw).

#### 2.4.3. Assay of Nitric Oxide (NO) Scavenging Activity

Nitric oxide scavenging activity was measured at 550 nm following the procedure described by Parul et al. [12]. Inhibition of nitrite formation by the cowpea seed extracts was calculated relative against a calibration curve of ascorbic acid. Results were expressed as mg of ascorbic acid equivalent per 100 g of dry seed weight (mg AAE/100 g seeds dw).

#### 2.4.4. Hydroxyl Radical Scavenging Activity (HRSA)

The scavenging activity of hydroxyl radical was measured at 532 nm following the procedure described by Perjési and Rozmer [13]. The HRSA of the seed extracts was reported as the percentage (%) of inhibition of deoxyribose degradation at the concentration of 50 mg/mL of dry seed weight.

#### 2.4.5. Lipid Peroxidation Inhibitory Assay

The method described by Jaishree et al. [14] was used to determinate the inhibition of lipid peroxidation potential of cowpea seed extracts at 532 nm. Results were expressed as inhibitory percentage (%) of lecithin peroxidation at the concentration of 50 mg/mL of dry seed weight.

### 2.5. Statistical Analysis

The results presented as mean ± Standard Deviation for triplicate analysis were subjected to one-way analysis of variance (ANOVA) followed by Tukey’s test. A *p* value < 0.05 was considered significant. The Pearson Correlation test was used to determine the correlation between the phenolic content, antioxidant and anti-lipid peroxidation activities, and the colour of the seeds. The statistical analysis was performed using XLSTAT version 7.5.2 (Addinsoft, Paris, France).

## 3. Results and Discussion

### 3.1. Phytochemistry Analysis: Phenolic Content of Cowpea Varieties

Table 1 shows the phenolic contents in the seeds of cowpea varieties from Burkina Faso. Significant differences were observed amongst the total phenolic and total flavonoid content values from different varieties of cowpea seeds.

The total polyphenol content of the different cowpea varieties varied widely from 692.03 ± 9.58 to 63.14 ± 4.45 mg GAE/100 g of seeds dw. The variety of cowpea TVU 14676 (coloured seeds) showed a significantly (*p* < 0.05) high content of total phenolic and the variety KVx 396-4-5-2D (white seeds) showed the lowest total phenolic content. The thirty-one cowpea varieties showed different capacities of the seeds to accumulate total phenolic content. Over a 10-fold variation in the total phenolic concentration of seeds was observed between the cowpea varieties.

The total flavonoid content of the cowpea seeds varied from 23.95 ± 0.21 to 7.46 ± 1.01 mg QE/100 g of seeds dw respectively for the varieties Mougne (coloured seeds) and KVx 780-1 (white seeds). The Mougne variety contained a significantly higher content of flavonoids among all the other cultivars with the exception of the Gorom local, Tiligré, CR06-07, Yiisyandé, KVx 745-11P, TVU 14676, Labagela local, Niizwé, and Gourgou varieties. Over a 3-fold germplasm variation was observed for total flavonoid content in the germplasm of cowpea varieties. According to Zhao et al. [7] and Moreira-Araújo et al. [3], the polyphenol variation observed in different studies can be due to genetic and environmental factors, variation between cultivars, and the extraction procedure used. Many studies have shown a positive correlation between phenolic content and health benefits [15]. The phenolic compound content of several legumes was studied and classified into three different groups (low: <100, medium: 100–200, high: >200 mg GAE/100 g) by Marathe et al. [16]. According to their classification system, the cultivar of cowpea TVU 14676 (692.03 ± 9.58 mg GAE/100 g of seeds dw) can be classified into the high phenolic compound content group. Most of the non-chlorophyll pigmentation of flowers, leaves, and seeds can be attributed to flavonoids, according to Awika et al. [6]. The large concentration of flavonoids in the seed impacts the seed coat colour and may directly influence the choice of cowpea varieties for food, as demonstrated by Awika et al. [6]. Previous epidemiological studies have suggested that dietary intake of flavonoid-rich foods can have a protective effect against human diseases associated with oxidative stress [2].

### 3.2. Antioxidant Activities

The antioxidant capacities of the different cowpea seed extracts, as determined by using several models involving different antioxidant mechanisms, are shown in Table 1. The abilities of the cowpea extracts to reduce iron (III) and to scavenge the radical DPPH (2,2-diphenyl-1-picrylhydrazyl), as well as nitric oxide and hydroxyl radicals, were evaluated.

The Variety KVx 745-11P (white seeds) showed high DPPH radical scavenging activity (10.38 ± 0.02 mg AAE/100 g of seeds dw) but not significantly higher than those of KVx 780-4, KVx 780-4, CR06-07, Kondèsyoungo, Komcallé, KVx 780-3, KVx 780-9, KVx 30-309-6G, and Gorom local genotypes. The Mougne (coloured seeds) variety showed the lowest DPPH scavenging activity (5.58 ± 0.57 mg AAE/100 g of seeds dw). The results showed 1.8-fold differences in free radical scavenging (DPPH) activity of cowpea seeds between the cowpea varieties. A DPPH assay based on an electron transfer reaction and hydrogen-atom abstraction was used to measure the reducing ability of antioxidants towards DPPH [17].

The abilities of the cowpea extracts to reduce iron from the form (III) to the form (II) showed a 12-fold genotypic variation between the cowpea varieties. The cultivar TVU 14676 (coloured seeds) had a significantly higher reducing power capacity (311.46 ± 0.82 mg AAE/100 g of seeds dw) followed by the cultivars Mougne, KVx 402-5-2, and IT 93K-693-2 (247.26 ± 0.82, 179.65 ± 4.31, and 160.49 ± 11.91 mg AAE/100 g of seeds dw, respectively). The variety Niango local showed weak reducing power capacity (25.51 ± 0.77 mg AAE/100 g of seeds dw). Iron, which is essential for life, is an extremely reactive metal and causes oxidative damage to lipids, proteins, and other cellular components [18].

The IT81 D-994 genotype (white seeds) showed the strongest nitric oxide radical scavenging activity (4.30 ± 0.39 mg AAE/100 g of seeds dw) while the IT 97K-489-3 cowpea variety showed the lowest nitric oxide radical scavenging activity (3.305 ± 0.13 mg AAE/100 g of seeds dw). The overproduction of nitric oxide (NO), a pro-inflammatory mediator involved in various physiological processes in the human body, can lead to tissue damage and the activation of pro-inflammatory mediators associated with acute and chronic inflammation [19,20]. Therefore, cowpea extract might be beneficial for treatment of the inflammatory response.

The KVx 745-11P variety of cowpea (white seeds) showed a significantly (*p* < 0.05) high hydroxyl radical scavenging potential (69.94% ± 0.87%), followed by the varieties KVx 414-22-2 (66.83% ± 0.20%) and Labagela local (65.59% ± 0.29%) at the concentration of 50 mg/mL of seeds dw. The hydroxyl radical scavenging power of the KVx 771-10G (Nafi) variety obtained the minimum activity (46.45 ± 0.1% at the concentration of 50 mg/mL of seeds dw) compared to the other studied varieties of cowpea. The OH radicals are the most highly reactive and endogenous radicals generated during aerobic metabolism, causing strand damages in DNA, potentially leading to carcinogenesis, mutagenesis, and cytotoxicity [18].

Biological reactions often produce free radical in the body that are partly associated with the etiology of cancers and other chronic diseases [21,22]. Furthermore, some cowpea varieties remarkably scavenge DPPH, hydroxyl, and nitric oxide radicals and elicit good ferric reducing attributes. The seeds of these cowpea varieties might be able to reduce the risk of the chronic disease.

As shown in Table 1, the inhibition potential of the lipid peroxidation of the seeds varies widely between the thirty-one varieties of cowpea. The inhibition of lipid peroxidation percentage of the cowpea varieties varied from 98.21% ± 0.12% to 2.43% ± 1.46% at the concentration of 50 mg/mL of seeds dw. The variety TVU 14676 (coloured seeds) possesses the significant high lipid peroxidation inhibition percentage followed by CR06-07 variety. The cultivar KVx 780-9 showed the lowest lipid peroxidation inhibition percentage. High genotypic variation (over of 40-fold difference) in anti-lipid peroxidation potential was observed in the seeds of these cowpea varieties. Reactive oxygen species such as hydroxyl radicals can extract a hydrogen atom from lipids in bio-membranes’ phospholipids, inducing lipid peroxidation and damaging tissues in the body [14,23]. The adducts of lipid peroxidation may generate the oxidation of biomolecules, such as DNA, proteins, and other lipids, resulting in cellular damage [14]. This has been observed in aging and stress related diseases [23]. Thus, the cowpea seeds with anti-lipid peroxidation potential could prevent aging and chronic disease.

### 3.3. Phenolics Contribution to the Antioxidants Activities

Pearson correlation matrix between the phenolic content and antioxidant and anti-lipid peroxidation properties is presented in Table 2.

Significant relationships between total phenolic content and ferric reducing ability (*r* = 0.954) and anti-lipid peroxidation (*r* = 0.616), and between ferric reducing ability and anti-lipid peroxidation (*r* = 0.64) were observed. However nitric oxide scavenging activity of cowpea was found not to be correlated to its total phenolic and total flavonoid content. The total flavonoid content was also found not to be related with DPPH and hydroxyl radicals scavenging activities in 31 cowpea varieties. Many studies have shown a positive correlation between total phenolic content and antioxidant activity of dietary plants [24]. Natural antioxidants can delay the lipid oxidation process in food products by acting as free radical scavengers, reducing agents, chelators of pro-oxidant metals, or as quenchers of singlet oxygen [25]. According to Zhang et al. [7], the reducing power, scavenging DPPH activity, and anti-lipid-oxidation ability of an antioxidant were not always positively correlated with each other. The seed colour of cowpea genotypes is presented in Table 1. Seven genotypes have coloured seeds and twenty-four have white seeds.

### 3.4. Seed Colour Contribution to the Antioxidant Activities and Phenolics Contents

**Figure 1:**
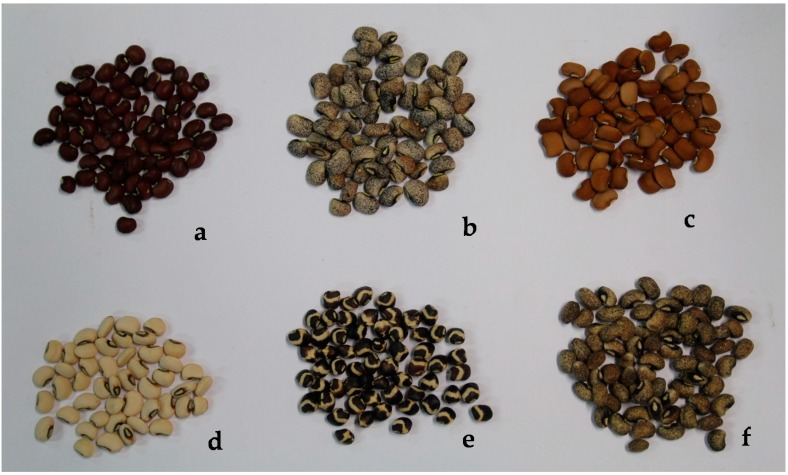
Seed colours of a few cowpea genotypes used. (**a**) CR06-07, (**b**) Local Labagela, (**c**) Gorom local, (**d**) Local Kondèsyoungo, (**e**) TVU 14676, (**f**) Mougne.

The seeds of cowpeas used in this study varied widely from white to black in colour. Six genotypes have brown coloured seeds, one has red coloured seeds, and twenty-four cowpea genotypes have white coloured seeds. The seeds of a few cowpea genotypes are shown in Figure 1.

The correlation coefficients between seed colour and other variables are presented in Table 2. There were strong positive significant correlations between the colour and the total phenolic content (*r* = 0.792, *p* < 0.001), the total flavonoid content (*r* = 0.348, *p* < 0.001), the ferric reduction ability (*r* = 0.721, *p* < 0.001), and the anti-lipid peroxidation potential (*r* = 0.481, *p* < 0.001). Coloured seeds of cowpea varieties possess higher total phenolic content, total flavonoid content, ferric reduction ability, and anti-lipid peroxidation activities in comparison to the colourless ones. Furthermore, a low correlation was also found between seed colour and % inhibition of hydroxyl radical (*r* = 0.391, *p* < 0.001) and nitric oxide radical scavenging capacity (*r* = 0.315, *p* < 0.001). In contrast, no correlation was observed between seed colour and the DPPH radical scavenging activity. Several studies suggest that the more pigmented cowpea cultivars had significantly (*p* < 0.05) higher total phenolic content, total flavonoid content, and antioxidant activities than the less pigmented cultivars [8].

## 4. Conclusions

In summary, our results clearly showed that wide variations in phenolic contents, as well as antioxidant and anti-lipid peroxidation activities, exist amongst the Burkina Faso cowpea varieties. Cowpea genotypes with coloured seed coats showed the highest phenolic content, ferric reduction ability, and anti-lipid peroxidation activities. This study provides important information for breeding cowpea varieties with health-enhancing attributes. The results generated from this study suggest that cowpea could contribute significantly in the management and/or prevention of degenerative diseases associated with free radical damage. However, further studies in different locations are needed in order to evaluate the environmental effect on phenolic content and antioxidant potential in the cowpea varieties used in this work. More research is also needed to characterize the phenolic compositions and structures that contribute to the health-enhancing potential of cowpea.

## Figures and Tables

**Table 1:** Phenolic content and antioxidant properties of cowpea genotypes.

Samples		Phytochemical Data		Antioxidant Powers				
Cowpea Genotype	Seed Colour	Total Phenolic Content (mg GAE/100 g of Seeds dw)	Total Flavonoid Content (mg QE/100 g of Seeds dw)	DPPH (mg AAE/100 g of Seeds dw)	FRAP (mg QE/100 g of Seeds dw)	Lipid Peroxidation Leucithine Inhibition (%)	Hydroxyl Radical Scavenging Activity (%)	NO (mg AAE/100 g of Seeds dw)
Gorom local	Brown	265.07 ± 16.56 ^c,d^	22.05 ± 1.87 ^a,b^	9.97 ± 0.12 ^a,b,c^	123.67 ± 0.71 ^e,f^	82.40 ± 0.62 ^b,c^	51.62 ± 0.23 ^k,l^	3.60 ± 0.1 ^b,c^
58-57	White	75.29 ± 11.16 ^j,k^	11.65 ± 0.30 ^i,j,k^	6.55 ± 0.18 ^i^	61.02 ± 0.19 ^m,n,o,p^	42.56 ± 0.87 ^q^	60.78 ± 0.41 ^c^	3.75 ± 0.37 ^a,b,c^
CR06-07	Red	250.84 ± 19.79 ^d^	20.86 ± 0.55 ^a,b,c^	10.18 ± 0.15 ^a,b^	128.41 ± 1.85 ^e^	83.05 ± 1.06 ^b^	58.75 ± 0.23 ^d^	3.62 ± 0.24 ^b,c^
IT 81 D-994	White	72.03 ± 4.13 ^k,l^	14.23 ± 1.42 ^e,f,g,h,i,j^	8.46 ± 0.02 ^f,g,h^	53.32 ± 0.39 ^p,q^	54.29 ± 0.81 ^m^	58.00 ± 0.58 ^d,e^	4.30 ± 0.39 ^a^
IT 93 K-693-2	Brown	293.96 ± 4.70 ^c^	17.84 ± 0.68 ^b,c,d,e,f,g^	9.93 ± 0.2 ^a,b,c,d^	160.49 ± 11.91 ^d^	76.61 ± 0.21 ^d^	48.22 ± 0.59 ^m^	3.35 ± 0.07 ^c^
IT 97 K-489-35	White	91.44 ± 5.1 ^g,h,i,j,k,l^	16.53 ± 0.14 ^b,c,d,e,f,g,h,i^	8.24 ± 0.71 ^g,h^	63.31 ± 0.12 ^m,n,o^	58.87 ± 0.37 ^i,j,k^	50.70 ± 0.51 ^l^	3.30 ± 0.13 ^c^
IT 97K-573-2 (Yiisyandé)	White	78.25 ± 22.38 ^j,k,l^	20.54 ± 1.00 ^a,b,c^	9.57 ± 0.2 ^a,b,c,d,e^	56.47 ± 2.27 ^o,p^	50.76 ± 1.26 ^o,p^	57.57 ± 0.2 ^d,e^	3.91 ± 0.34 ^a,b,c^
IT 98K-205-8 (Niizwé)	White	80.47 ± 5.87 ^j,k,l^	19.98 ± 1.65 ^a,b,c,d,e^	9.76 ± 0.14 ^a,b.c.d.e^	100.22 ± 1.87 ^g^	65.88 ± 1.26 ^f,g,h^	56.46 ± 0.54 ^e,f,g,h^	3.85 ± 0.11 ^a,b,c^
Kondèsyoungo local	White	127.88 ± 7.57 ^f,g,h^	17.33 ± 2.05 ^b,c,d,e,f,g,h,i^	10.13 ± 0.19 ^a,b^	90.83 ± 2.95 ^h,i^	67.02 ± 0.75 ^f^	52.96 ± 1.37 ^j,k^	3.62 ± 0.11 ^b,c^
KVx 30-309-6G	White	87.44 ± 1.68 ^i,j,k,l^	10.66 ± 2.57 ^j,k^	10.04 ± 0.00 ^a,b^	55.79 ± 1.46 ^o,p^	41.77 ± 0.25 ^q^	55.12 ± 0.46 ^h,i^	3.73 ± 0.17 ^a,b,c^
KVx 396-4-5-2D	White	63.14 ± 4.45 ^l^	13.24 ± 0.66 ^f,g,h,i,j,k^	8.43 ± 0.26 ^f,g,h^	45.05 ± 1.1 ^q^	51.79 ± 1.43 ^n,o^	55.38 ± 0.43 ^f,g,h^	3.56 ± 0.11 ^c^
KVx 402-5-2	Brown	480.03 ± 5.3 ^b^	15.02 ± 0.43 ^c,d,e,f,g,h,i,j^	9.16 ± 0.06 ^b,c,d,e,f,g^	179.65 ± 4.31 ^c^	71.60 ± 0.33 ^e^	51.82 ± 0.70 ^k,l^	3.59 ± 0.18 ^b,c^
KVx 414-22-2	White	74.70 ± 5.15 ^j,k,l^	16.73 ± 0.18 ^b,c,d,e,f,g,h,i^	9.19 ± 0.45 ^b,c,d,e,f^	58.19 ± 0.78 ^n,o,p^	49.21 ± 0.69 ^p^	66.83 ± 0.20 ^b^	3.63 ± 0.03 ^b,c^
KVx 421-2J	Brown	186.84 ± 20.50 ^e^	14.59 ± 0.30 ^d,e,f,g,h,i,j^	8.88 ± 0.37 ^d,e,f,g,h^	73.03 ± 3.67 ^k,l^	53.51 ± 0.45 ^m,n^	48.38 ± 0.54 ^m^	3.42 ± 0.11 ^c^
KVx 442-3-25-SH (Komcallé)	White	130.25 ± 14.77 ^f,g^	13.95 ± 7.94 ^f,g,h,i,j^	10.13 ± 0.16 ^a,b^	84.22 ± 0.29 ^i,j^	65.24 ± 0.12 ^f,g,h^	57.64 ± 0.25 ^d,e^	3.75 ± 0.05 ^a,b,c^
KVx 61-1	White	164.33 ± 5.73 ^e,f^	16.77 ± 1.65 ^b,c,d,e,f,g,h,i^	9.90 ± 0.18 ^a,b,c,d^	79.20 ± 1.25 ^j,k^	81.76 ± 0.54 ^b,c^	61.50 ± 0.32 ^c^	4.20 ± 0.24 ^a,b^
KVx 65-114	Brown	268.33 ± 7.18 ^c,d^	13.87 ± 0.07 ^f,g,h,i,j^	8.80 ± 1.25 ^e,f,g,h^	97.52 ± 1.72 ^g,h^	60.44 ± 0.43 ^i^	47.60 ± 0.26 ^m,n^	3.44 ± 0.10 ^c^
KVx 745-11P	White	114.55 ± 1.28 ^g,h,i,j^	20.38 ± 2.37 ^a,b,c,d^	10.38 ± 0.02 ^a^	62.35 ± 0.65 ^m,n,o^	64.23 ± 0.21 ^h^	69.94 ± 0.87 ^a^	3.66 ± 0.05 ^b,c^
KVx 771-10G (Nafi)	White	103.59 ± 10.97 ^g,h,i,j,k^	17.56 ± 0.99 ^b,c,d,e,f,g,h^	9.45 ± 0.16 ^a,b,c,d,e,f^	86.76 ± 0.9 ^i,j^	58.15 ± 0.12 ^j,k^	46.45 ± 0.1 ^n^	3.90 ± 0.13 ^a,b,c^
KVx 775-33-2G (Tiligré)	White	107.14 ± 3.59 ^g,h,i,j,k^	21.93 ± 0.94 ^a,b^	9.58 ± 0.22 ^a,b,c,d,e^	68.88 ± 1.63 ^l,m^	55.58 ± 0.33 ^l,m^	60.71 ± 0.67 ^c^	3.80 ± 0.09 ^a,b,c^
KVx 780-1	White	100.03 ± 2.24 ^g,h,i,j,k,l^	7.46 ± 1.01 ^k^	9.90 ± 0.29 ^a,b,c,d^	62.54 ± 3.71 ^m,n,o^	66.60 ± 0.21 ^f,g^	56.89 ± 0.72 ^e,f,g^	3.75 ± 0.21 ^a,b,c^
KVx 780-3	White	105.81 ± 0.93 ^g,h,i,j,k^	12.37 ± 0.79 ^g,h,i,j,k^	10.12 ± 0.12 ^a,b^	66.28 ± 1.95 ^l,m,n^	57.44 ± 0.25 ^k,l^	56.79 ± 0.26 ^e,f,g,h^	3.85 ± 0.32 ^a,b,c^
KVx 780-4	White	124.47 ± 9.58 ^f,g,h,i^	14.11 ± 1.00 ^f,g,h,i,j^	10.22 ± 0.33 ^a,b^	83.98 ± 1.95 ^i,j^	64.59 ± 0.86 ^g,h^	57.05 ± 0.40 ^e,f^	3.74 ± 0.15 ^a,b,c^
KVx 780-6	White	106.25 ± 19.12 ^g,h,i,j,k^	13.48 ± 0.94 ^f,g,h,i,j^	9.96 ± 0.2 ^a,b,c^	68.94 ± 0.18 ^l,m^	65.45 ± 0.37 ^f,g,h^	55.25 ± 0.17 ^g,h,i^	3.84 ± 0.31 ^a,b,c^
KVx 780-9	White	114.55 ± 35.44 ^g,h,i,j^	11.73 ± 0.25 ^h,i,j,k^	10.08 ± 0.10 ^a,b^	63.47 ± 0.72 ^m,n,o^	2.43 ± 1.46 ^r^	60.61 ± 0.97 ^c^	3.80 ± 0.31 ^a,b,c^
Labagela local	White	190.70 ± 21.13 ^e^	20.06 ± 0.50 ^a,b,c,d,e^	9.93 ± 0.15 ^a,b,c,d^	118.87 ± 0.98 ^f^	60.68 ± 0.22 ^i^	65.59 ± 0.29 ^b^	3.69 ± 0.05 ^a,b,c^
Mougne	White	470.25 ± 5.43 ^b^	23.95 ± 0.21 ^a^	5.58 ± 0.57 ^i^	247.26 ± 0.86 ^b^	80.69 ± 0.45 ^c^	47.33 ± 0.37 ^m,n^	3.84 ± 0.07 ^a,b,c^
Moussa local	White	78.40 ± 2.04 ^j,k,l^	18.00 ± 0.55 ^b,c,d,e,f,g^	9.71 ± 0.16 ^a,b,c,d,e^	63.49 ± 0.27 ^m,n,o^	53.72 ± 0.45 ^m,n^	53.25 ± 0.25 ^j,k^	3.53 ± 0.06 ^c^
Niango local	White	73.07 ± 4.38 ^k,l^	11.93 ± 0.66 ^h,i,j,k^	8.93 ± 0.08 ^c,d,e,f,g,h^	25.51 ± 0.77 ^r^	60.37 ± 0.66 ^i,j^	57.31 ± 0.28 ^d,e^	3.80 ± 0.24 ^a,b,c^
TVU 14676	Brown	692.03 ± 9.58 ^a^	20.10 ± 0.96 ^a,b,c,d^	7.97 ± 0.18 ^h^	311.46 ± 0.82 ^a^	98.21 ± 0.12 ^a^	52.40 ± 0.54 ^j,k^	3.80 ± 0.07 ^a,b,c^
TZ-1 (Gourgou)	White	89.36 ± 7.40 ^h,i,j,k,l^	18.99 ± 1.50 ^a,b,c,d,e,f^	9.69 ± 0.04 ^a,b,c,d,e^	55.98 ± 1.69 ^o,p^	54.86 ± 0.87 ^m^	53.58 ± 0.20 ^i,j^	3.68 ± 0.10 ^b,c^

Values are expressed as mean values ± standards deviation (*n* = 3 independent experiments). Means with different superscript letters along the row differ significantly (*p* < 0.05). GAE: Gallic Acid Equivalent; QE: Quercetin Equivalents; DPPH: 2,2-diphenyl-1-picrylhydrazyl; AAE: Ascorbic Acid Equivalent; FRAP: Ferric Reducing Antioxidant Power; NO: Nitric Oxide.

**Table 2:** Pearson correlation coefficient.

	(1)	(2)	(3)	(4)	(5)	(6)	(7)	(8)
Total phenolic (1)	1	**0.340**	**−0.348**	**0.954**	**0.616**	**−0.381**	−0.101	**0.792**
Total flavonoid (2)		1	−0.099	**0.446**	**0.430**	−0.028	−0.010	**0.348**
DPPH (3)			1	**−0.367**	−0.080	**0.286**	−0.024	0.203
FRAP (4)				1	**0.640**	**−0.366**	−0.057	**0.721**
LPO Inhibition (5)					1	**−0.257**	−0.012	**0.481**
HRS (6)						1	**0.261**	**0.391**
NO (7)							1	**0.315**
Colour of seeds (8)								1

Values in bold indicate a significant value (*p* < 0.05).
